# Patterns and determinants of pathways to reach comprehensive emergency obstetric and neonatal care (CEmONC) in South Sudan: qualitative diagrammatic pathway analysis

**DOI:** 10.1186/s12884-017-1463-9

**Published:** 2017-08-29

**Authors:** Khalifa Elmusharaf, Elaine Byrne, Ayat AbuAgla, Amal AbdelRahim, Mary Manandhar, Egbert Sondorp, Diarmuid O’Donovan

**Affiliations:** 1 Graduate Entry Medical School, University of Limerick, Limerick, Ireland; 2 Reproductive & Child Health Research Unit (RCRU), University of Medical Sciences & Technology, Khartoum, Sudan; 3 Institute of Leadership, Royal College of Surgeons in Ireland, Dublin, Ireland; 4 Family, Women’s and Children’s Cluster, WHO, Geneva, Switzerland; 5 Royal Tropical Institute, Amsterdam, Netherlands; 6 National University of Ireland Galway, Galway, Ireland

**Keywords:** EmONC, Referral system, Maternal mortality, Conflict affected fragile states, South Sudan, Quality of care, Competency, Access

## Abstract

**Background:**

Maternity referral systems have been under-documented, under-researched, and under-theorised. Responsive emergency referral systems and appropriate transportation are cornerstones in the continuum of care and central to the complex health system. The pathways that women follow to reach Emergency Obstetric and Neonatal Care (EmONC) once a decision has been made to seek care have received relatively little attention. The aim of this research was to identify patterns and determinants of the pathways pregnant women follow from the onset of labour or complications until they reach an appropriate health facility.

**Methods:**

This study was conducted in Renk County in South Sudan between 2010 and 2012. Data was collected using Critical Incident Technique (CIT) and stakeholder interviews. CIT systematically identified pathways to healthcare during labour, and factors associated with an event of maternal mortality or near miss through a series of in-depth interviews with witnesses or those involved. Face-to-face stakeholder interviews were conducted with 28 purposively identified key informants. Diagrammatic pathway and thematic analysis were conducted using NVIVO 10 software.

**Results:**

Once the decision is made to seek emergency obstetric care, the pregnant woman may face a series of complex steps before she reaches an appropriate health facility. Four pathway patterns to CEmONC were identified of which three were associated with high rates of maternal death: late referral, zigzagging referral, and multiple referrals. Women who bypassed nonfunctional Basic EmONC facilities and went directly to CEmONC facilities (the fourth pathway pattern) were most likely to survive. Overall, the competencies of the providers and the functionality of the first point of service determine the pathway to further care.

**Conclusions:**

Our findings indicate that outcomes are better where there is no facility available than when the woman accesses a non-functioning facility, and the absence of a healthcare provider is better than the presence of a non-competent provider. Visiting non-functioning or partially functioning healthcare facilities on the way to competent providers places the woman at greater risk of dying. Non-functioning facilities and non-competent providers are likely to contribute to the deaths of women.

**Electronic supplementary material:**

The online version of this article (10.1186/s12884-017-1463-9) contains supplementary material, which is available to authorized users.

## Background

Worldwide 300,000 maternal deaths occur every year [1]. Maternal death is “the death of a woman while pregnant or within 42 days of termination of pregnancy, irrespective of the duration and site of the pregnancy, from any cause related to or aggravated by the pregnancy or its management, but not from accidental or incidental causes” [2]. A maternal near miss is when “a woman who nearly died but survived a complication that occurred during pregnancy, childbirth or within 42 days of termination of pregnancy” [3]. Maternal mortality is a good indicator of the availability of health services. A peak in maternal mortality occurs during the intrapartum period around childbirth and the first day post-partum [4]. Hence Filippi et al., in the Lancet series on maternal survival [5], called for a clear strategic vision that prioritises the intrapartum period in order to reduce maternal mortality. The main reasons for maternal deaths are the lack of skilled birth attendants, remoteness of health facilities in relation to catchment area, delays in referral for emergency obstetric care, and poor implementation of interventions at the facility level [4, 6]. Responsive emergency referral systems, clear referral protocols and appropriate transportation at the first level of care are cornerstones in the continuum of care and a crucial part of the health system to ensure timely and appropriate transfer to comprehensive emergency obstetric and neonatal care (CEmONC).

There is growing evidence to show the profound negative impact of conflict on maternal mortality: populations that have experienced armed conflict have among the highest rates. O’Hare et al. [7] compared the adjusted maternal mortality ratio of 21 African countries that have experienced recent armed conflict with 21 African countries that have not. They found that the median adjusted maternal mortality is significantly higher in countries with recent conflict (1000 per 100,000 births) compared to countries that had not had such conflict (690 per 100,000 births; *p* = 0.005) [7].

The social determinants of health and related social rules and taboos in South Sudan have all been affected by the conflict [8, 9]. Women in South Sudan face alarmingly low maternal health status as indicated by the high maternal mortality ratio (2037 per 100,000 live births) [10]. These women have very little control over reproductive decisions and have been exposed to sexually transmitted infections and unwanted pregnancies [9]. The war placed high pressure on them to reproduce as a national obligation [11]. South Sudanese women are caught in a vicious cycle of high fertility and high child mortality leading to high maternal morbidity and mortality [12]. Utilisation of maternal healthcare services is very low; less than 20% of women receive four antenatal visits during their pregnancy, and less than 20% of deliveries are attended by skilled birth attendants. The caesarean section rate is 0.6% [10, 13–15]. Less than 4% of women use family planning: the most common method is the lactation amenorrhea [8, 16].

Maternity referral systems have been under-documented, under-researched, and under-theorised [17]. The pathway that women follow to reach EmONC, once a decision has been made to seek care, has received relatively little attention [18]. The research presented in this article identifies such patterns and determinants of pathways pregnant women follow from their homes once a decision to seek help during labour has been made until they reach an appropriate health facility.

### Context: South Sudan

South Sudan has suffered from civil conflict for most of the time since the independence of Sudan in 1956. More than two million people are estimated to have died since 1956, and more than four million were internally displaced or became refugees. A comprehensive peace agreement was signed in 2005, ending the civil war. South Sudan gained independence in 2011. Since new internal conflicts in December 2013, the country has experienced repeated eruptions of violence and political instability.

Health services were very limited in southern Sudan during the colonial period (1898-1956) when missionaries provided most of the formal health care [19]. During the civil wars, health services in southern Sudan were provided and controlled by official government authorities in Khartoum and were restricted to a few major ‘garrison towns’. Health services in the area controlled by the rebel Sudan People’s Liberation Army / Movement (SPLA/M) were largely provided through humanitarian channels by Non-Governmental Organisations (NGOs), Faith Based Organisations (FBOs) and UN agencies. In the late 1990s, the SPLA/M created a secretariat of health and a department for relief operations to oversee the provision of health services in areas under their control [20, 21]. As a result of the comprehensive peace agreement in 2005 a decentralisation policy was adopted in which the main aim was to devolve power to a hierarchy of local authorities [22].

Currently (2017), the Ministry of Health in South Sudan operates through a decentralised structure operating at four levels: community, county, state and central. Services are delivered through the following: Primary Health Care Units (PHCUs), Primary Health Care Centres (PHCCs), County Hospitals (CH) and State Hospitals (SH) / Teaching Hospitals (THs) [23]. The Community level services (PHCUs and PHCCs) implement the Basic Package of Health Services (BPHS) which consist of interventions to improve maternal and child health and nutrition, and control communicable and non-communicable diseases [24]. The Ministry of Health proposed that each PHCU would have one professional midwife and four medical assistants; and each PHCC would have three professional midwives, one health visitor, four medical assistants, ten professional nurses, one pharmaceutical technologist and two laboratory technologists. [25] Several of PHCUs and PHCCs are currently not functioning in this way [23]. Challenges include poor coordination and unclear lines of responsibility between the levels of the health system and stakeholders [26]. Most of the existing health facilities are in poor condition and are inadequately equipped, with minimal operational capacity and scarce human resources including physicians (latest reported rate of 1 per 65,574 population) and midwives (1 per 39,088 population) [27]. In addition, the general infrastructure, such as roads and telecommunication systems, are weak, with access further impeded by insecurity [28].

South Sudan’s Reproductive Health Strategic Plan 2013 – 2016 provides a national framework for the promotion and implementation of reproductive health programmes and delivery of services in South Sudan. The strategic plan proposes the integration of Reproductive Health Services as one of the components of the BPHS and the implementation of the basic and comprehensive EmONC as the foundation for universal access to health under the BPHS. However, the strategic plan indicated clearly the limited number of facilities and the shortage of human resources to manage obstetric and neonatal emergencies [25]. The South Sudan EmONC needs assessment in 2013 indicated that South Sudan had only 10 functioning BEmONC and 14 CEmONC facilities with an estimated deficit of 77 BEmONC and 8 CEmONC facilities [29, 30].

## Methods

### Study population and setting

This research is part of a study that was conducted from 2010 to 2012 in Renk County to gain an in-depth understanding of access to maternal healthcare in South Sudan to inform local policy and practice [31–33]. Renk County is one of the 13 counties that constitute the Upper Nile State in South Sudan. It is located in the northern part of the state, close to the international border with Sudan. In the April 2010 census, the population of Renk County was 137,751 with 29,589 women of reproductive age (15–49 years) [34].

### Study design

This qualitative research used two data collection methods: *Critical Incident Technique (CIT)* and *stakeholder interviews*.

#### Critical incident technique (CIT)

CIT was used to study incidents of maternal death (MD) and maternal near miss (MNM) cases that occurred within the past two years, to ensure the interviewees could still recall details of the incident and to minimise recall bias. CIT involves the use of a set of procedures to collect in-depth data on human behaviour and people’s experiences with regard to significant incidents [35], which in this study were MD and MNM cases. The method was used, through a series of in-depth interviews with witnesses or those involved, to systematically identify pathways to healthcare during labour, determinants and behavior associated with the events. CIT was conducted with selected events among women of different ages, parity and ethnic backgrounds in order to develop an understanding of their day-to-day activities and habits; how they perceive reproductive and family health in general with a focus on maternal health, labour and the challenges that arise resulting in maternal mortality or near miss. Their responses reflected culture, norms and beliefs providing a comprehensive overview to help understand the factors influencing their behaviours and decisions. These stories of maternal mortality and morbidity reflect the reality of community and health systems that shape maternal and infant care in a country affected by years of war.

#### Stakeholder interviews

After conducting the CIT interviews, the research team conducted face-to-face interviews with relevant maternal health stakeholders. In this study, the term ‘stakeholders’ refers to individuals from different institutions and agencies that hold relevant official positions, or are perceived as having a role to play or a perspective on maternal health in Renk County [36, 37].

### Sampling and participants

#### Critical incident technique (CIT)

A critical case purposive sampling approach was employed to identify MD and MNM cases. Critical case sampling has been defined as a process whereby, “individuals, groups, or settings are selected that bring to the fore the phenomenon of interest such that the researcher can learn more about the phenomenon than would have been learned without including these critical cases” [38]. The few first events were selected purposively through village midwives and midwives working in Renk hospital who identified one or more events of MD or MNM. After one event was identified, a snowball technique was used to identify the other cases, by asking the interviewees to identify other ‘critical cases’ among their social networks including neighbours or relatives, who had a similar experience. Thirteen critical incident cases were identified and included in this study, of which five were MD and eight were MNM cases.

#### Stakeholder interviews

A purposive sampling technique was used to identify, approach and recruit potential key informants. Following this, a snowball sampling technique was applied to identify and recruit further interviewees. Senior officers who hold relevant official positions in Renk County government and county health department were approached. Health personnel at Renk hospital, Jalhak health centre and Geiger health centre, who were involved in providing the service to pregnant women either directly or indirectly, were also invited to participate. Relevant NGOs, FBOs, and community and religious leaders were also identified. Other relevant key informants who were identified during the data collection stage were also included in the study. In total, 28 interviewees were identified and invited to participate (Table 1), all of them accepted and were included.

**Table 1:** Stakeholders interviewed

Local government (2)	County health department (4)	Healthcare providers (14)	Employees of NGOs (2)	Employees of FBOs (3)	Community and religious leaders (3)
· County executive director · Head of Humanitarian Aid Commission	· A senior manager at County Health Department · A senior manager at HIV and Sexually Transmitted Diseases Department · A senior manager at Reproductive Health and Midwifery Department · A senior manager at the vaccination programme	· Hospital manager (1) · Doctors (3) · Health visitor (1) · Hospital Midwives (3) · Nurses (2) · Medical assistant (1) · Village Midwives (2) · Traditional Birth Attendant (1)	· Mubaderon · Mercy Corp	· The Organisation of Invitation to Islam · Church organisation · Camboni Missionary School	· Imam · Church father · Village chief

### Data collection

#### Critical incident technique (CIT)

After identifying a MD or MNM case, an appointment was made for the interviews when informed consent was verbally obtained. The interviews were scheduled at a time and venue chosen by each interviewee. KE, AAA, and AAR conducted the interviews in Arabic and Juba-Arabic (the local language). In each case, semi-structured interviews were conducted with all available witnesses of the critical case including the woman’s husband, mother, in-laws, sisters, the traditional birth attendant (TBA), the midwives and, in MNM cases, the woman herself. Some of the interviews were done on a one-to-one basis, where each respondent was interviewed separately and in other cases a group interview was conducted, depending on the preferences of the people involved. In the latter case, one participant in the group would give an overview of the event, after which each of the participants would be interviewed according to the described sequence of events. During the interview the researcher began by reintroducing the study, followed by ice-breaking questions and getting to know the interviewee. This led to personal and demographic data, background information about the family, details about the respondent and their relationship to the deceased mother in case of maternal deaths being collected. The main question was ‘what happened?’ The interviewee was enabled to speak about the event as much as possible. Then the researcher returned to the beginning of the story and began asking follow-up and probing questions in order to get as much detailed data as possible to understand the care seeking pathways. Depending on the specific event and interviewee, questions were also asked regarding past obstetric history, previous similar experiences, culture, beliefs and health-seeking behaviours. Interviews ended with questions as regards future resolutions, recommendations if any, and revisiting the answers of some questions that were not clearly answered. Interviewees were thanked for their full participation and help (Additional file 1).

#### Stakeholder interviews

Once a stakeholder was identified, an appointment was made for the interview. The interviewees chose the time and venue. At the beginning of each interview, the researcher explained the purpose of the study and obtained verbal informed consent. The interviews were video or audio recorded and notes were taken during the interviews in a diagrammatic style (i.e. with key words and phrases recorded and linked). Although the research team prepared pre-planned questions to ask during the interview, they allowed questions to flow naturally, based on the respondent’s position and information provided. At the start of the interviews, participants were briefed and informed about their right to refuse to answer any question, which none chose to do. Then they were asked for background information and introductory questions about issues facing women in South Sudan. Following this, more focused questions were asked about issues related to acceptability, affordability, accessibility, availability and quality of healthcare. The interviews concluded with questions about their recommendations to improve women’s health in South Sudan (Additional file 2).

### Data analysis

#### Critical incident technique (CIT)

Data generated from each case, comprising all interviews related to the event, was transcribed and translated into English. All transcripts related to each case were read through completely to familiarize the researcher with the content. A narrative was developed by summarising each event from all its related transcripts. The narrative was mapped by illustrating on paper the path of each incident that the patient followed from the onset of labour pains or of complications until she reached the final health facility. The mapping identified actors, decision makers, decision points, consequences, timeframe and geographical locations. This diagrammatic pathway analysis was done by the lead author (KE). This pathway analysis was then examined by other members of the research team (EB and DOD) to add to the rigor of the analysis. This method of analysis gave new insights into the data and helped to identify decision-making processes, delays in accessing healthcare, and referral patterns.

#### Stakeholder interviews

Thematic inductive analysis was used to analyse data generated from stakeholder interviews. This identified key themes and analytic questions requiring further exploration. This approach was used to ensure that the identified themes were data-driven, without trying to fit it into a pre-existing coding frame. The five phases suggested by Braun and Clarke [39] were used for thematic analysis. These include data management and familiarisation, initial coding, identification of themes, reviewing themes, and defining and naming themes. NVivo 10 was used to manage, code and analyse data.

### Ethical consideration

The field research team consisted of the principal researcher (KE) and two research fellows (AAA and AAR) who were fully trained in advance of field work on dealing with sensitive topics. They were trained to pause during the interviews before dealing with particularly sensitive issues and remind participants of the option not to respond. Safeguarding measures were applied for psychological safety of people interviewed. The research team had to build trust and rapport with the families through visiting their homes several times. The first visit was spent sitting and talking with the family to explain the study and to sympathise with their pain and loss. Death is a sensitive issue, surrounded by many negative circumstances and incidents. Discussing this with relatives often brought back a wave of negative emotions. The interviewers had to probe sensitively, steer the interview so that it stayed focused, and at all times consider the interviewee’s comfort. Any cues or signals by which the interviewee was indicating distress were carefully observed. The interviewer gave the interviewees time to express significant emotion and advised them on relevant support services in the area to consult if they experienced distress either during or after the interview. Follow up visits were also arranged to meet with the family members.

## Results

**Fig. 1:**
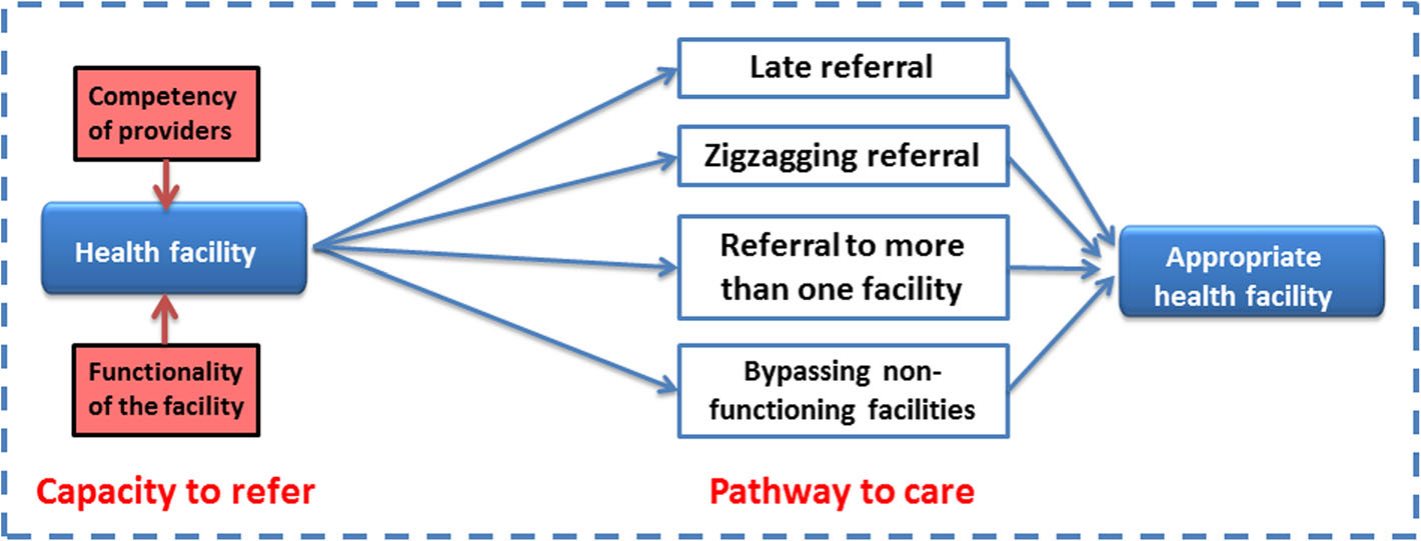
Pathways to reaching an appropriate healthcare facility that provide CEmONC

Based on the analysis of the critical incident cases and stakeholders’ interviews four patterns of pathway to reach CEmONC were identified: (1) Late referrals to appropriate facilities, (2) Zigzagging referral, (3) Multiple referrals, and (4) Bypassing non-functioning healthcare facilities (Fig. 1). The main causes of the identified MD and MNM were bleeding (2MD & 3MNM), eclampsia (1MD & 2 MNM) and obstructed labour (2MD & 3 MNM). However, underpinning all the pathways was the functionality of BEmONC facilities and competency of their health providers.

### Functionality of BEmONC

Depending on the geographical location, the nearest healthcare facility at the level of the community for a pregnant woman could be a village PHCU serviced by a professional midwife or a nurse, or a PHCC serviced by a medical assistant. Women with emergency obstetric conditions (such as severe bleeding or convulsions) seek help at these rural facilities. When PHCCs cannot manage emergency cases, they refer women to the County Hospital (CH) in Renk city which provides CEmONC. However, Renk hospital at the time of the study did not have a blood bank, and staff relied on family members to donate blood. Failure to secure a blood donor usually resulted in the family being referred to Rabak hospital or Kusti hospital in White Nile state in North Sudan, where blood bank facilities were available, but this was not feasible for everyone referred.

According to a senior manager at Renk County’s health department, each village in Renk County should have an appropriate number of PHCCs, to implement the basic package services and ensure community participation. A senior manager at the Reproductive Health and Midwifery Department in Renk County stated that each health centre in the county should provide BEmONC and should have at least one trained midwife to provide antenatal care and delivery, yet most of the centres have only a medical assistant or a male nurse. Thus PHCCs were not in a position to provide a supportive system linked to Renk hospital as a referral hospital if CEmONC was required.

The health facilities in the county are not evenly distributed geographically. For example, there are more than 15 villages in Shomodi Payam in the south east area of Renk town, all of them are served by only one pharmacy and two health units. Some areas have no health facilities at all. On the other hand, most of the functioning healthcare facilities are concentrated in Renk town. There are two centres for antenatal care around Renk town. The midwifery school manages the first one, which is part of Renk hospital. The second one is attached to and run by the Episcopal Church.

*“There is no planning for distribution of the health facilities. Facilities are built according to the available fund and what people want. In the Catholic church in Renk there are 20 midwives and one of them is the head of the midwifery services. They play an important role in providing midwifery services in the Renk city. Pregnant women call them to come to their house to help them with delivery.” (Employee in a FBO)*

According to a senior manager of the county’s vaccination programme, many of the health centres in villages are not functioning and most of the services are provided by Renk hospital. In the periphery of Renk Payam (Geiger, Jalhak and Shomodi), there are 16 PHCUs and PHCCs, but 8 of them (50%) are not functioning. Additionally, the working environment in the poorly functioning PHCUs and PHCCs is not suitable for providing an acceptable level of health service:

*“They come to me. I help as much as I can, but sometimes I don’t have medications. If there is something I can’t handle I take her to the medical assistant. If we had a doctor, we would not face such problems.” (Trained midwife in Jalhak)*

The health centre in Jalhak, 80 km south of Renk town, was built in the 1980s. Before the war, the maternal health services in the health centre were provided by a doctor, midwives and nurses. However, due to the war, the centre was closed and its functions were never fully restored. It now provides very limited health services at a level of a health unit. There are also small private clinics, laboratories and pharmacies that are owned and run by health assistants and nurses:

*“This health centre in Jalhak was there since the 1980s. There was a doctor who treated people for free. Before the war, there were not too many health problems. After the beginning of the war, people fled the area and the health centre was just a building. After the comprehensive peace agreement in 2005, some people tried to work at the centre but could not because they didn’t live here originally. They were from other places and didn’t know how to manage the problems. Their salaries didn’t arrive on time and sometimes not at all.” (Medical assistant in Jalhak)*

Absenteeism of health workers was also raised as a main concern in the health centres. For example, some stakeholders indicated that a medical assistant in one of the peripheral health centres was not available most of the time because he worked in a private centre in Renk town. Another concern raised was the presence of unqualified personnel. The example was given of a person who worked in the pharmacy of one the villages who was not a qualified pharmacist, but who treated people for malaria and eye infections based on the peoples’ complaints.

### Competency of birth attendants

The ability of birth attendants to know whether a patient needed to be referred to another facility influences maternal survival. Birth attendants can be trained midwifes or TBAs. The competency of these cadres varies.

#### Competency of TBA

The TBA is also called ‘the rope midwife’, because she uses a rope, which she fixes to the roof of the house or to a tree so that the delivering woman can hold it to help her in the delivery. TBAs claim that they inherit experience from their mothers and grandmothers. One TBA said that she got her experience from a dream. She dreamed about a woman who was in labour and asked her to help; she took a razor blade, cut the woman, and delivered a healthy baby to a healthy woman. TBAs do not use scissors, but prefer razor blades, which they buy from the market. They consider a new razor to be sterilised. They wrap one side with a cloth, and use the other side to cut the woman. They use straight sewing needles to suture the cuts. A senior manager at the Reproductive Health and Midwifery Department in Renk County complained about the ability of the TBAs to deal with complicated deliveries. It is difficult to convince TBAs to come and stay for one year in the midwifery school for training. Most of the TBAs refuse to stay in the town for a long time as they do not want to leave their families and husbands.

*“In the past, TBAs have arrived to Renk hospital with pregnant women with their babies partly delivered; parts of the foetus, such as the head, the arm or the leg, outside the woman’s body and the rest of the body still inside.” (A senior manager at the Reproductive Health and Midwifery Department)*

Many other stakeholders also indicated that TBAs have inadequate skills to manage labour. They stated that a TBA might give advice if there is no trained midwife available. The TBA’s role is to reassure the woman in labour about the baby and her health and to provide advice on taking rest, not doing strenuous housework. According to A senior manager at the Reproductive Health and Midwifery Department, the TBA cannot identify the severity or the magnitude of certain maternal and neonatal complications, or the appropriate actions to take. The TBA, after exhausting all her efforts, may call the trained midwife if the delivery is not progressing well. The trained midwife will take over and if the midwife fails to solve the problem, the woman should be referred to the hospital.

TBAs, however, feel that they have enough experience to do their job and some TBAs do not see the need for training. They know that people around them trust them and listen to their advice. TBAs claim that if they failed to manage a case and it became complicated, they would refer the woman to “those who are more experienced” than them in the hospital. Some TBAs expressed the desire to have access to certain drugs for use during pregnancy and labor, with some indicating the need for supportive training to enable them to use appropriately. The drugs that they mentioned are those ‘used to treat anaemia’, ‘stop bleeding during labour’, and ‘local anaesthesia’.

#### Competency of midwives

The training of midwives takes place in two midwifery schools in the Upper Nile state, one in Renk town and one in Malakal city. The midwifery school in Renk town contacts the executive director of each payam (local administrative division) to request community leaders to nominate women from their localities for midwifery training. Attendants are required to be aged between 26 and 39 years. They complete a one-year training course in order to be become a qualified midwife. There are two types of trained midwives: a nurse midwife who graduates without a ‘midwife’s medical bag’ because she will work in the hospital, and a village midwife who will be based in the community and graduates with a ‘midwife’s medical bag’, or ‘suitcase’, hence she may be called a ‘suitcase midwife’. She is also called the ‘legal midwife’, to differentiate between her and ‘the illegal’ non-skilled TBAs.

The midwifery training curriculum in Renk midwifery school commences with basic instructions on instruments like scissors and forceps, and how to use and sterilise them. The course includes how to sterilise cotton and gauze, how to clean the midwife suitcase, and arrange the equipment inside it. Students are taught about ‘dry labour’, including drugs used during labour, cervix measurements and how to determine the level of cervical dilatation during labour. Then they are trained on ‘wet labour’, including how to cut using a scissors, and how to deliver and hold the baby and placenta. Each student must perform ten deliveries before graduating. After the training course, the trained midwives are recruited by their county councils to work in villages. Their monthly salary is about SDG 210 (USD 70). Recently the state government dismissed many of midwives recruited in this way and stopped their salaries due to budget limitations. The Director of Reproductive Health and Midwifery in Renk County acknowledged that the midwives who trained in these schools contributed to improving maternal health, because they know when to refer the pregnant women to hospitals. Funding to operate the midwifery school is the big challenge facing the sustainability of the training.

**Fig. 2:**
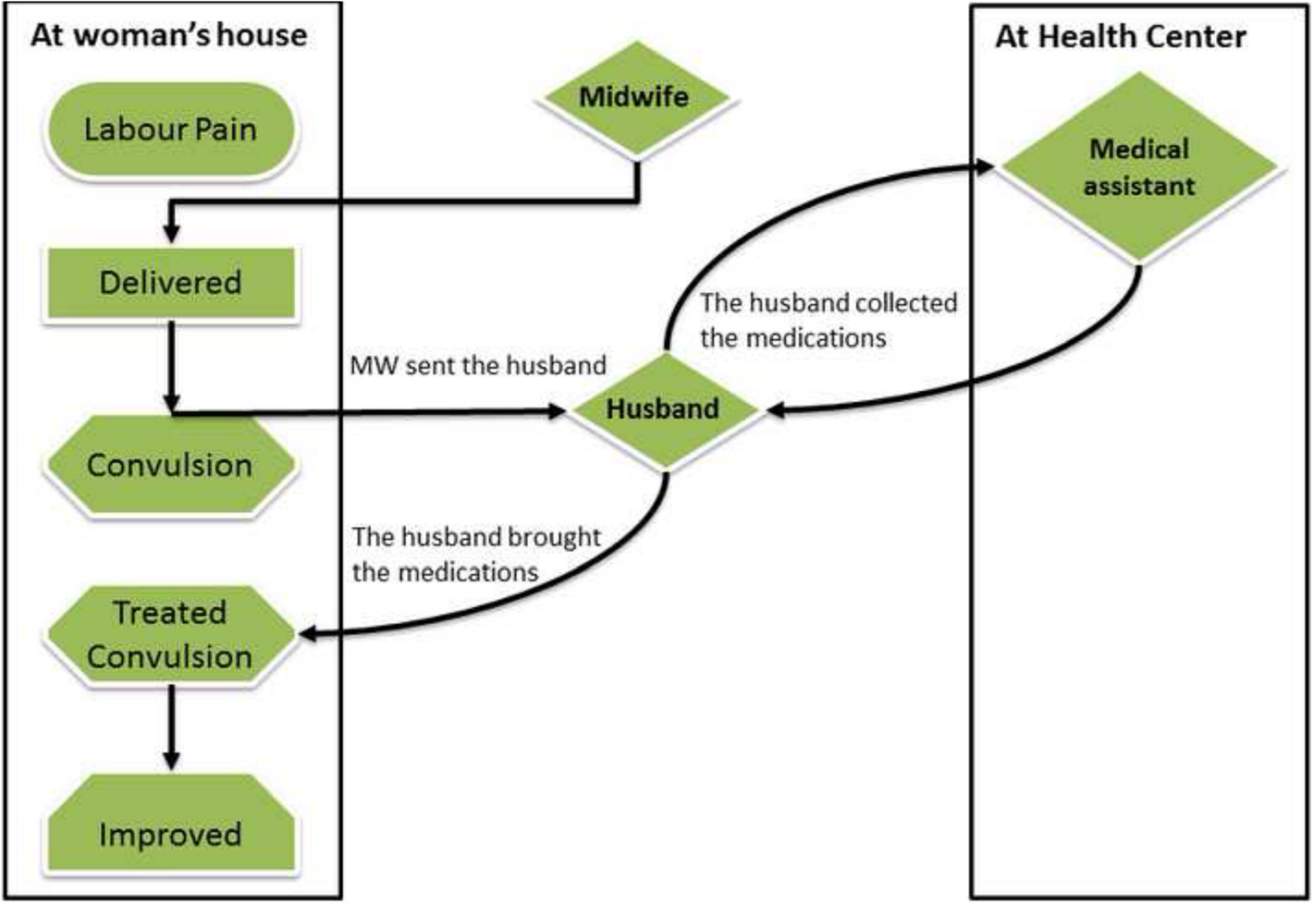
Competency of birth attendants. She experienced post-partum eclampsia. Her eyes rolled and she salivated excessively. The trained midwife immediately inserted a spoon into her mouth to prevent her from biting her tongue. She sent the husband running to collect infusions and injections from the nearest health centre. The husband went to the centre and described the situation to the medical assistant, who gave him IV fluids and injections to take them back to the midwife to treat the convulsion. The convulsions continued for about five minutes until the midwife gave the treatment. The woman recovered and did not have another fit. Five hours later she was nursing her new born baby. (7JMNM)

Competency of the birth attendant in crucial decision making emerged as a major influence on women accessing appropriate healthcare. One of the critical incident cases described showed that when a midwife sent the husband to the health centre to collect medication, the patient survived. If she had tried to refer her to Renk hospital the woman might have died. This is illustrated in Fig. 2.

### Pathways to care

#### Late referrals to appropriate facilities

Late referrals to appropriate facilities were reported when TBAs were reluctant to refer women before acknowledging problems with the labour. The TBAs may have assured the pregnant woman that everything would progress well and that the delivery would be very smooth. The TBAs try to manage the delivery as much as they can. The TBAs tend to keep the women in labour with them for long periods. When the duration of labour exceeds two to three days, or if one of the two main danger signs (bleeding or convulsion) occurs, TBAs declare that they can no longer manage the labour. Sometimes, the father and pregnant woman might notice that the labour is not progressing well and decide to declare the TBA’s failure before she does so herself. The referral process is delayed until failure is declared, either by the TBA or family member. Two maternal death cases illustrate the variety of reasons that led to a late referral to appropriate healthcare facilities:

*She had four children. Her family called for the TBA when her labour pains began. The TBA waited until the third day to ask the family to take her to the hospital. The family and pregnant woman went to the nearest rural hospital in Melut village. The woman presented to the casualty department at 8:00 am. The doctor examined her to discover that she had a ruptured uterus. (10RMD)*

*She was a very young primigravida living in a village on the outskirts of Palouge (170 km south of Renk town). When she was in labour, her family called for the TBA to come to help deliver her baby. Day after day passed until she became restless, febrile and no progress had been made with the labour. They decided to seek professional healthcare on the fourth day. (11RMD)*

**Fig. 3:**
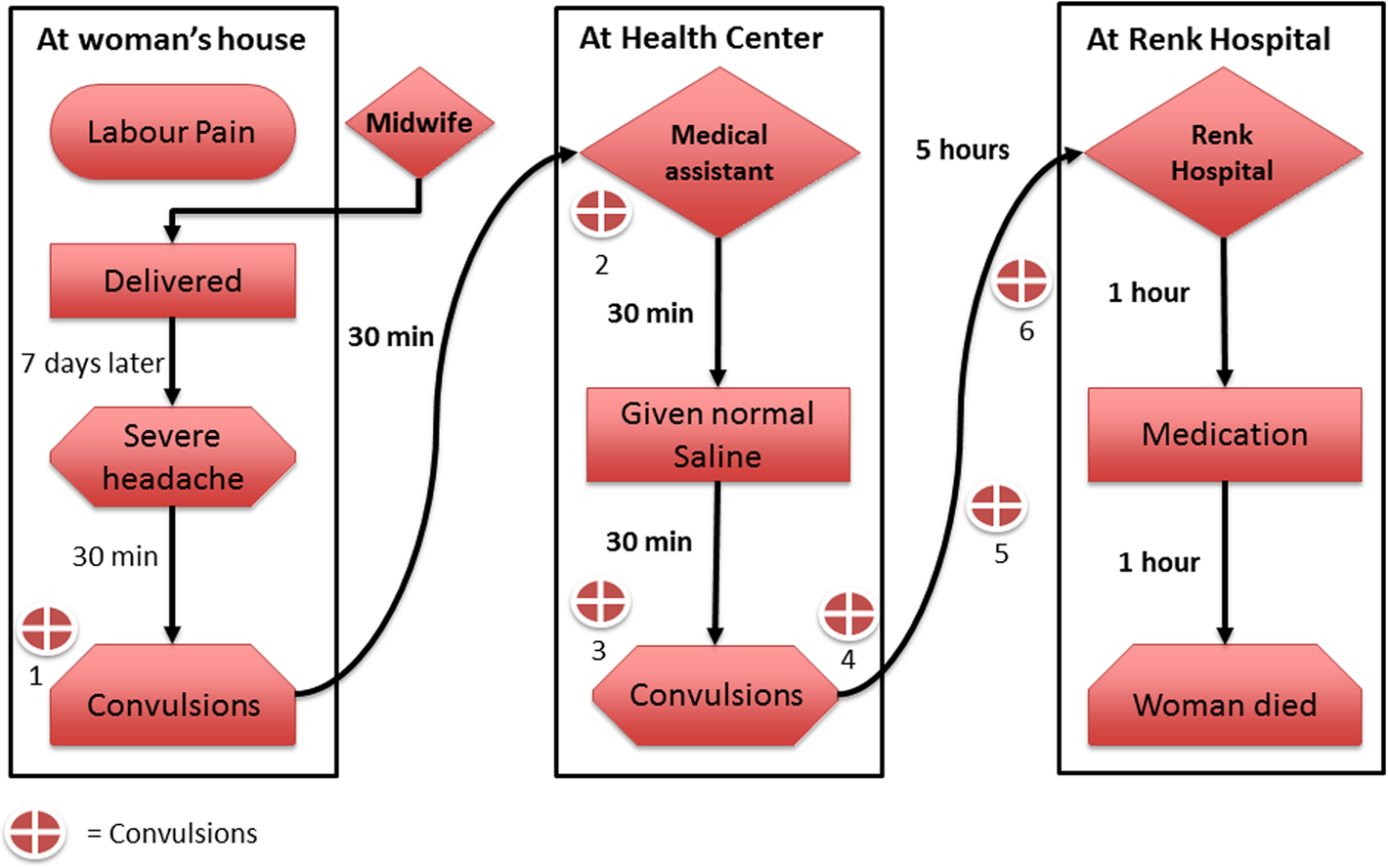
Late referrals to appropriate facilities. She had post-partum eclampsia. She had a fit and lost consciousness for approximately a minute. Her mother quickly inserted a spoon in her mouth and took her to the nearby health centre for help. On arriving she had another fit. The medical assistant in charge gave her a normal saline infusion without checking her blood pressure: this led to another fit. Her mother took the infusion off and decided to take her daughter to Renk hospital. On their way to the market on the same donkey-driven cart that took them to the health centre, another fit occurred. Luckily they found a pickup truck and could afford to rent it. On the way the pregnant woman experienced two fits. They arrived at Renk hospital four hours later. Unfortunately, after two hours in the hospital the pregnant woman died in her mother’s arms, despite the doctor’s efforts. (6JMD)

Another case was a mother who tried to manage the situation herself and a medical assistant in the health center failed to manage the case effectively and to reassure the family. The family ended up taking the decision themselves to transfer the critically ill patient to Renk hospital, but they arrived too late for the doctors to save her. Figure 3 illustrates this case.

#### Zigzagging referral

**Fig. 4:**
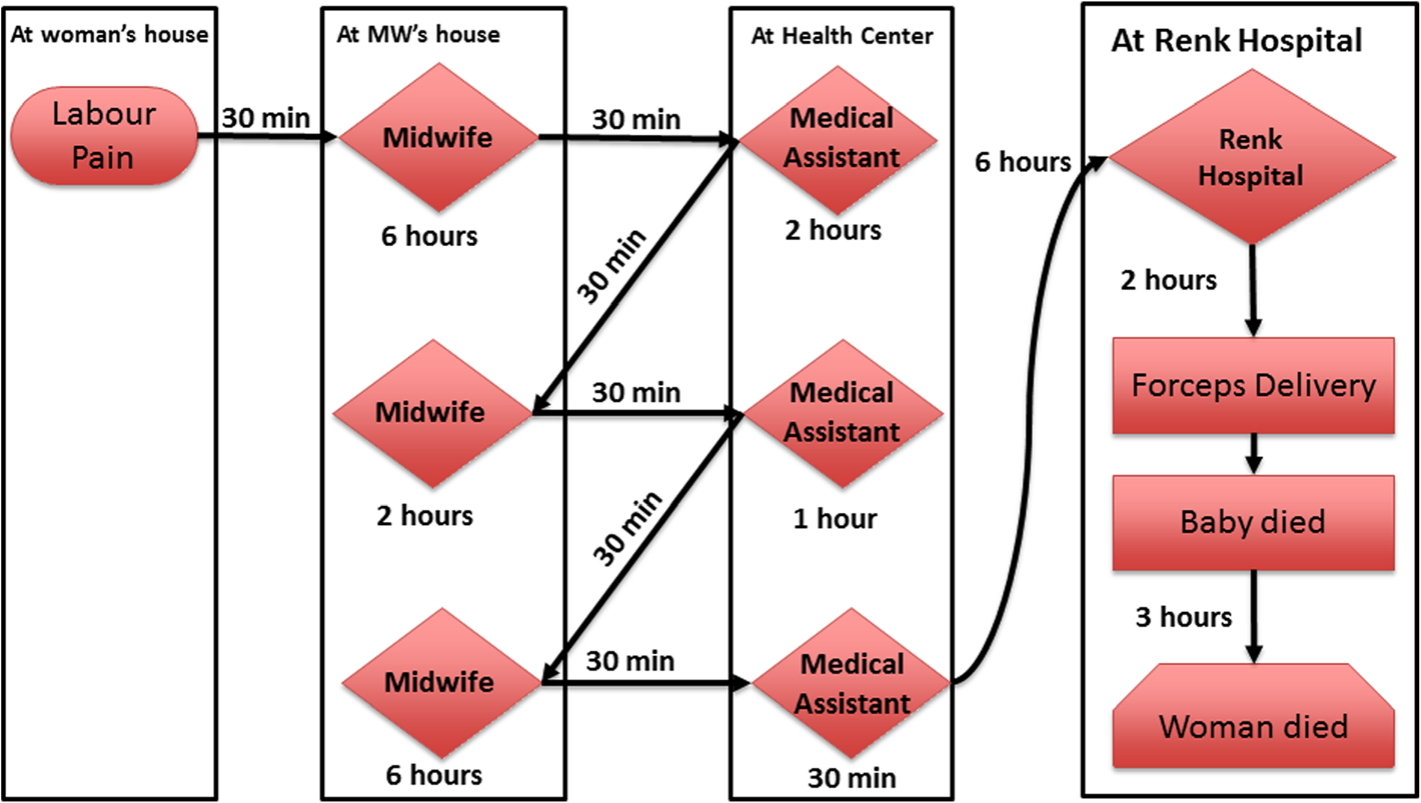
Zigzagging referral pattern. She was living in a village 80 km south of Renk town. The labour pains started at 8:00 am. After six hours, the trained midwife advised her family to take her to the medical assistant in the village health centre. Family members lifted the pregnant woman onto a donkey-driven cart and went to the village’s medical assistant. When they arrived, her water broke. The medical assistant prescribed drugs and told them that she was in labour and that the midwife should deliver her straight away. He sent them back to the midwife for delivery. After spending three hours with the midwife without progress, the pregnant woman was exhausted. The midwife advised them to go back to the medical assistant. They spent most of the night going back and forth between a midwife and a medical assistant until the midwife insisted on the medical assistant referring them to Renk hospital. They were referred shortly before 4 am – 20 h after labour had started. The pregnant woman’s father-in-law sought transportation to Renk hospital. He negotiated with the petroleum company to help them to get to the hospital. He waited until the driver arrived at 6.30 am and by 7 am they were on the road to Renk. At 10:00 am they arrived at the maternity ward of Renk hospital. All attempts to induce labour in the hospital failed and forceps had to be used. The baby was stillborn. Three hours later the mother died (5JMD)

The second pathway pattern identified is the ‘zigzagging referral’ that occurs when a delivering woman is referred back and forth between two healthcare providers. Each provider refers her to the other after failing to manage the labour, both failing to refer her to an appropriate facility. Such a case is illustrated in Fig. 4.

#### Multiple referrals

**Fig. 5:**
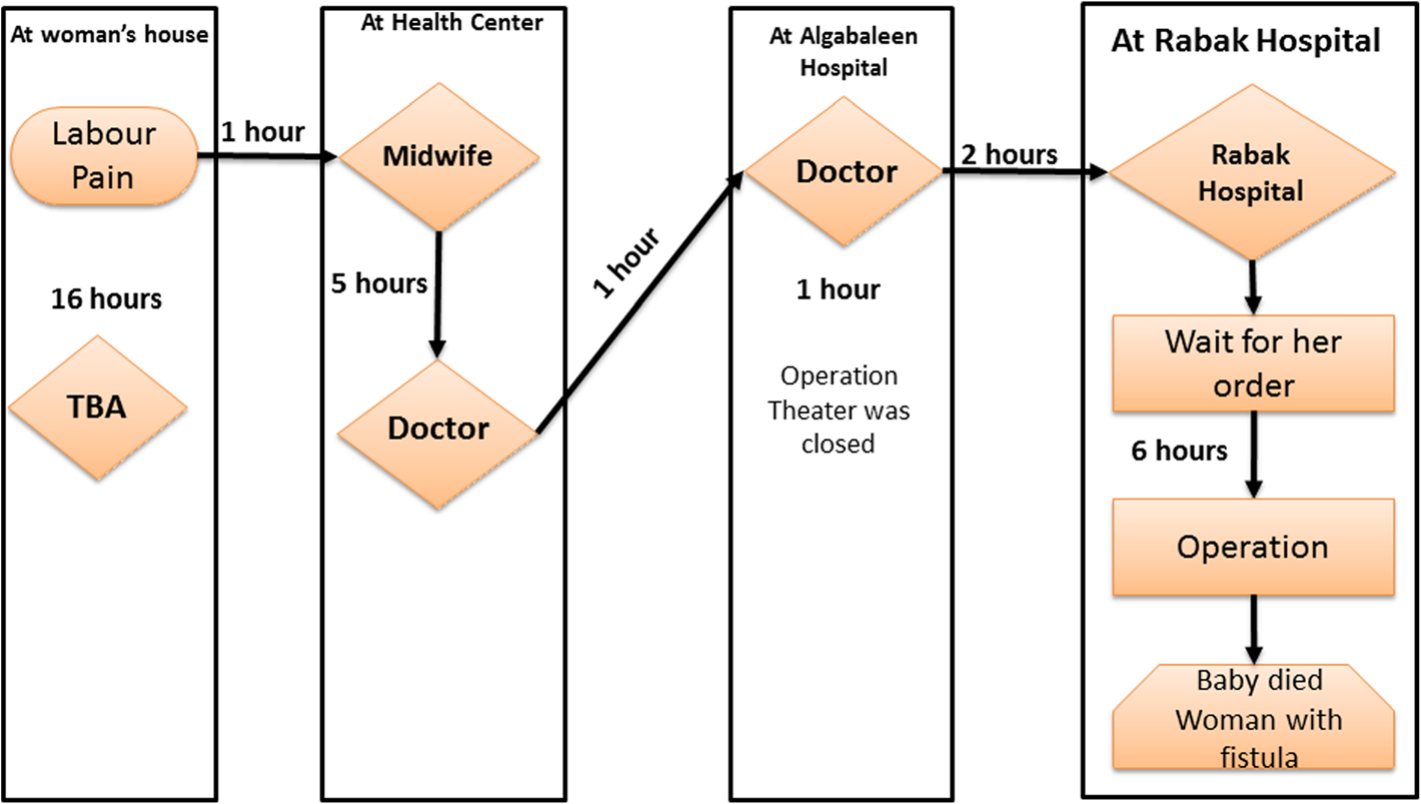
Multiple referral pattern 1. She was from a village 50 km north of Renk town. Her labour pains started at around 8 pm. Her family called the TBA who assured her that the delivery would be smooth, but the pregnant woman remained in severe pain for hours. By noon next day, she was in severe pain with no progress made. Her husband decided to go to the nearest health facility. They rented a pickup truck at a cost of USD10. They arrived at 1 pm at the house of the trained midwife, who was busy with another delivery. The midwife examined her and tried to deliver her. After a while she called for the doctor. The doctor referred them to Algabaleen hospital in North Sudan for an emergency caesarean section. At 6 pm they rented an ambulance, which cost them USD20 and crossed the border of North Sudan to Algabaleen town. They arrived at 7 pm and found that the theatre was closed for 72 h. They called another ambulance at 8 pm, which cost them another USD20. They arrived at Rabak city hospital at 10 pm. They were asked to pay USD20 for the caesarean section operation. There were many patients ahead of them and she was not operated until 4 am. She was transfused the next day with 4 units of blood and was given medication. She was hospitalised for 22 days. During her stay, her family stayed with her and supported her as much as they could. Her husband sold their cattle to cover the expenses. The expenses exceeded USD350. The woman lost her baby and lives now with a fistula. She goes every 14 days to the nearest health centre to her village to change her urine catheter. (13RMNM)

**Fig. 6:**
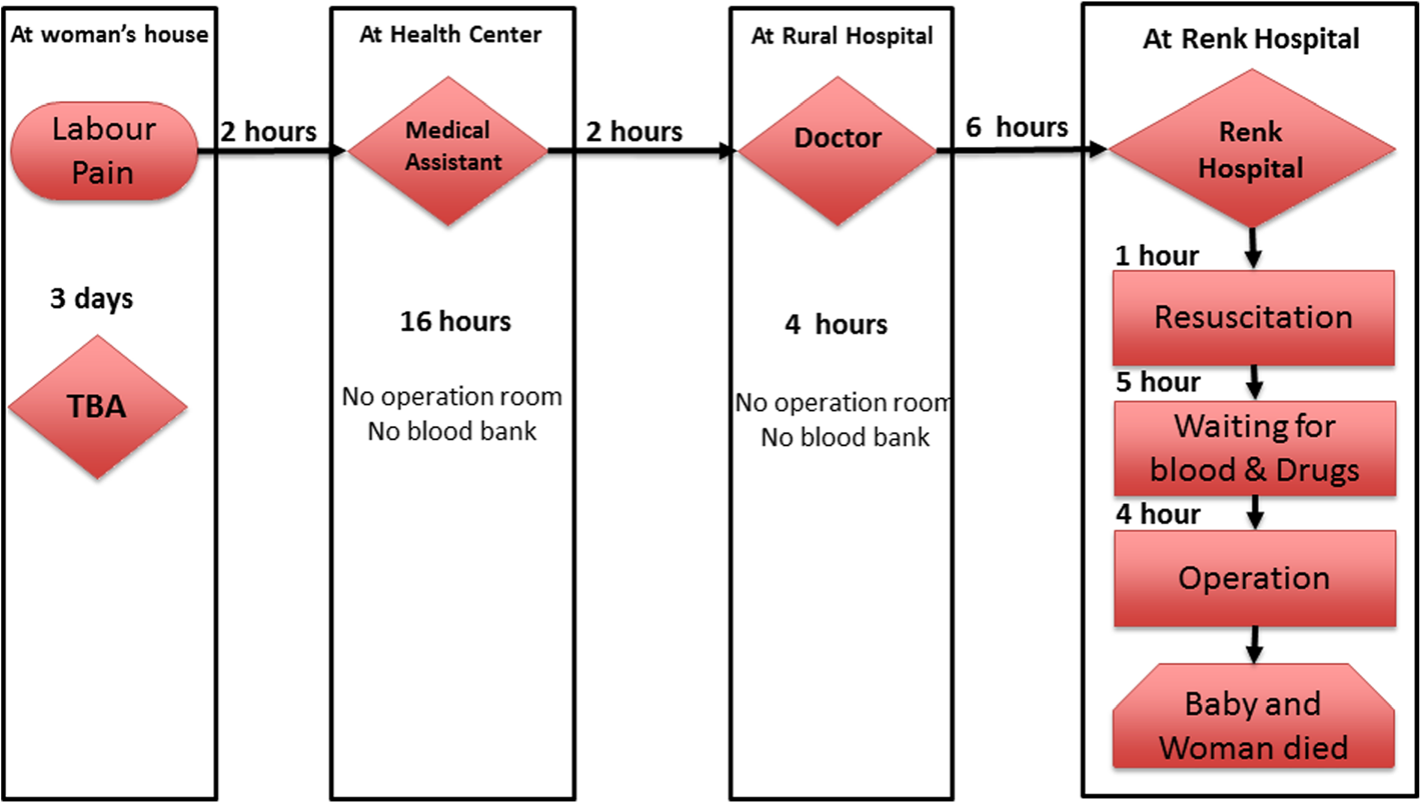
Multiple referral pattern 2. She was a very young primigravida living in a village 170 km south of Renk town. Her family called for the TBA to come to deliver her when her labour began. Day after day passed until she became restless, febrile and with no progress. They decided to seek professional healthcare on the 4th day. They arrived to the nearest health centre at 8 pm. The health centre is run by a medical assistant and a village midwife but does not have an operation room or blood bank. The medical assistant referred them to the rural hospital. On the 5th day at 1 pm the medical assistant called the doctor in charge of the rural hospital to inform him about the case and requested an ambulance for the referral. Unfortunately, the ambulance was not available. The road between the two towns is a dirt road that deteriorates during the rainy season. An hour later they managed to transfer the patient to the rural hospital that did not have an operating room or blood bank. The patient was only given antibiotics and referred to Renk hospital for an emergency caesarean section. At 11 pm the ambulance arrived at the maternity ward of Renk Hospital. The patient was given antibiotics and prepared for an emergency caesarean section. Due to delays in preparation and blood donation, the patient was only ready for the operation by 12 noon. The woman passed away during the operation. (11RMD)

**Fig. 7:**
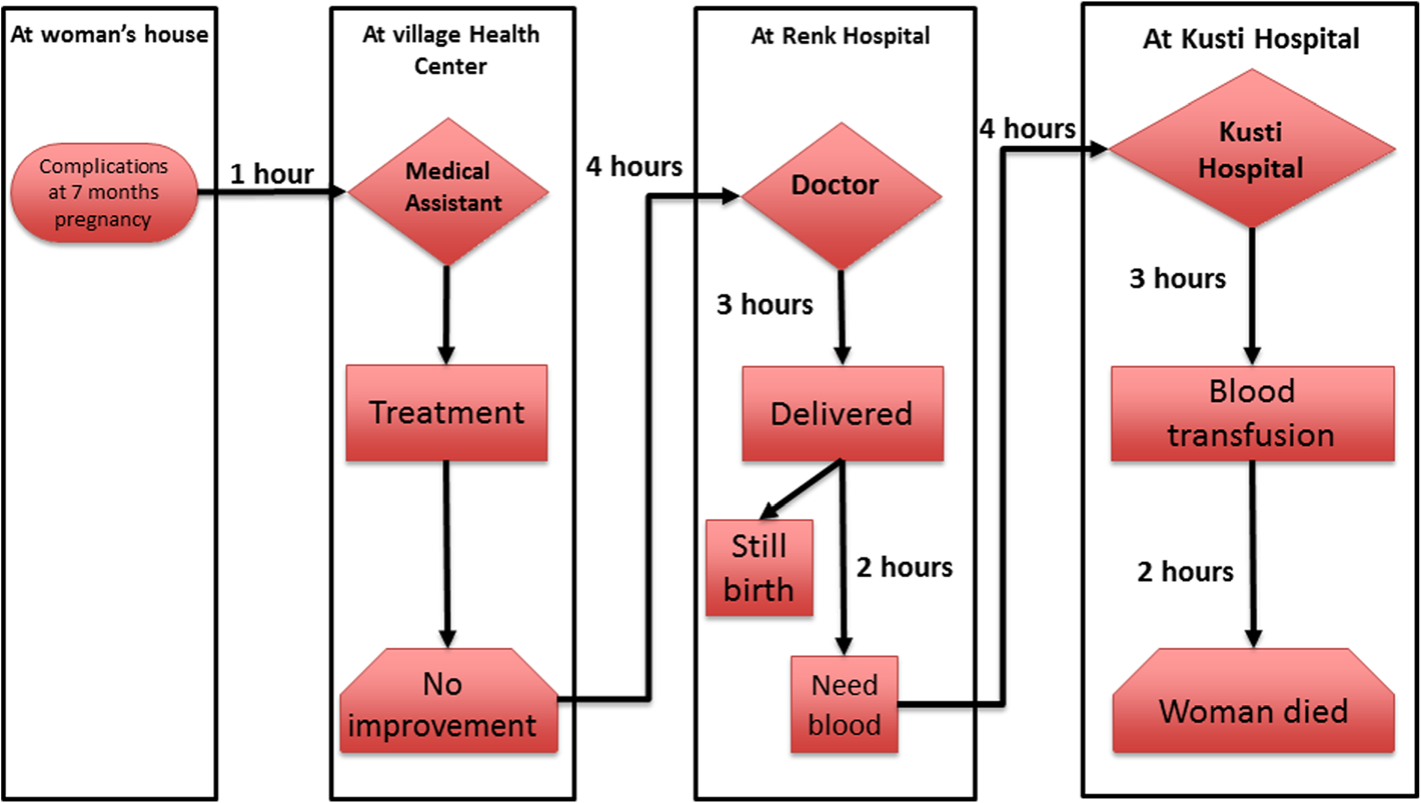
Multiple referral pattern 3. She was in her seventh month of pregnancy. She complained of body swelling and fever and was taken by her husband to the village health centre. She was given treatment but experienced no improvement in her condition. Her husband decided to take her to Renk hospital. In the hospital, she had labour pains and after a few hours she gave birth to a stillborn boy. The woman was devastated and severely depressed. She stopped eating and drinking, and was fed by intravenous fluids and administered various pills and injections. Her health continued to deteriorate. Her abdomen became uncomfortably distended. The doctors recognised that she needed a blood transfusion. As the blood bank in Renk hospital was closed the woman had to be taken to the nearest hospital in Kusti. Her husband realised he could not afford the transport costs. Some well off relatives sent him money when they heard about her condition. They arrived at Kusti hospital, where she was admitted and transfused on the same day. Unfortunately, an hour and a half later she passed away. The husband and her brother-in-law carried her body back and buried her in their village. (4JMD)

Another referral pattern is where the patient visits several healthcare facilities before reaching the appropriate facility able to provide CEmONC. Women seek help at the nearest health centre and are often referred to a ‘non-functioning’ rural facility (without appropriate staff), from where they might in turn be referred to another ‘non-functioning’ rural hospital, before reaching Renk hospital. Renk hospital should be able to provide a caesarean section and blood transfusion if needed. However, Renk hospital might refer patients on again to another referral hospital (such as Kusti hospital) due to lack of blood, or inability to perform operations. Three cases illustrate this pattern of multiple referrals (Figs. 5, 6 and 7).

#### Bypassing non-functioning healthcare facilities

**Fig. 8:**
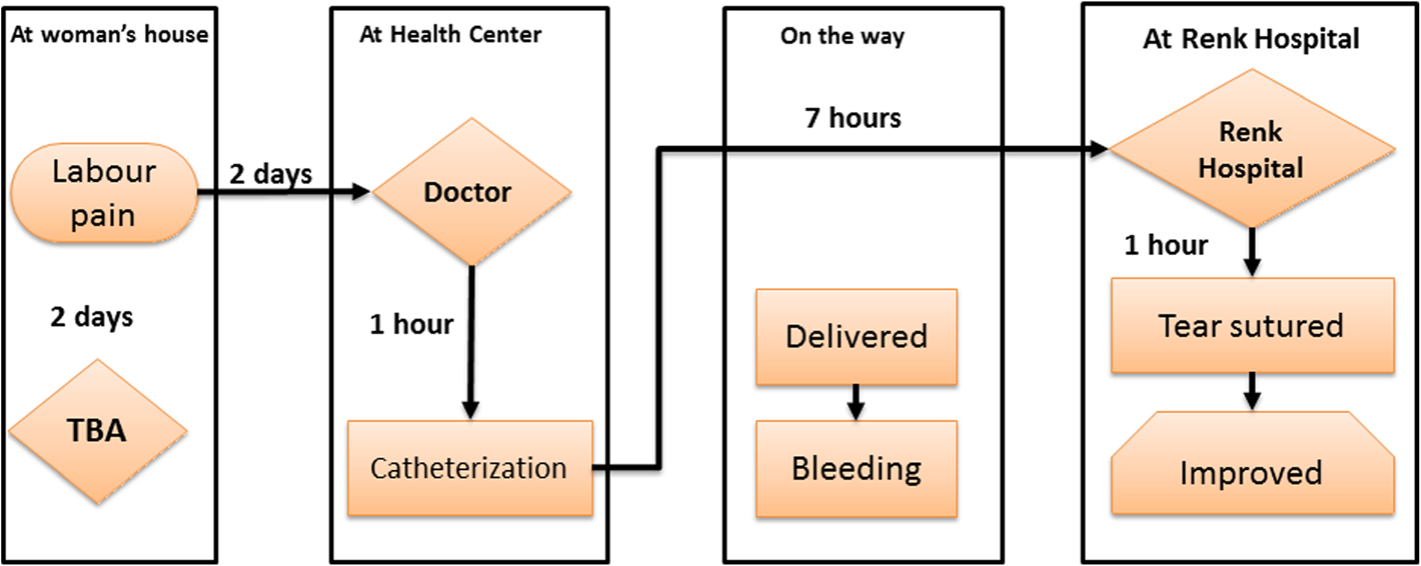
By-passing facilities. She lives in a village 250 km south of Renk town, more than 7 h’ drive from Renk hospital. The woman was in labour for 2 days with a TBA caring for her. On the third day, her family decided to seek medical help. They went to the nearest health center. The doctor the health centre inserted a catheter into her and referred them directly to Renk hospital. They were lucky to find a petroleum company pickup truck to bring them to Renk hospital. Midway, while the driver was driving at high speed to get them to Renk, the woman started to deliver the baby. They stopped the truck and she gave birth. The baby was alive, but the woman started to bleed. They continued driving to Renk. When they arrived at the hospital, the woman was lying restlessly, soaked in her blood, her baby beside her. She had a tear that was managed by the doctor and she was discharged 3 days later. (12RMNM)

The fourth pattern of referral involved bypassing non-functioning healthcare facilities. This emerged in the case of a woman who experienced prolonged labour and bleeding. The woman survived because she bypassed these non-functioning facilities and were instead referred directly to Renk hospital. Figure 8 illustrates this case.

## Discussion

Most studies on the utilisation of maternal health services focus on individual characteristics and behaviours that shape their engagement with services. The implicit assumption is that individuals are rational and autonomous in making decisions if they have sufficient information and knowledge about the outcome of courses of action they might take. Far less attention has been given to population’s engagement with health systems and the features of the community health systems that shape their choices [40].

Thaddeus and Maine [41] developed a framework, (later adapted by Gabrysch & Campbell [42]), to study delays in accessing maternal healthcare that focuses on the interval between the onset of an obstetric complication and its outcome [42]. The framework describes three phases of delay. The first is the delay in deciding to seek care on the part of the individual, the family, or both: this phase might be influenced by socioeconomic and cultural factors. The second phase is the delay in reaching an adequate healthcare facility: physical accessibility factors could influence this. The third phase is the delay in receiving adequate care at the facility: quality of care is the main influencing factor in this delay, and it also influences the other phases [41]. Our analysis of the pathways to care focus largely on phase two of this model.

Our findings show that the ‘second delay’ does not end with the woman reaching any healthcare facility. Reaching a non-functioning health facility could mark the beginning of a new cycle of the three delays. Our findings also show that the ‘second delay’ is influenced by quality of care including functionality of facilities and competencies of their providers to provide adequate healthcare at home and at the BEmONC facility. Therefore, the second phase of ‘reaching the healthcare facility’ should be described as ‘reaching an appropriate healthcare facility’ and may involve accessing more than one healthcare facility before an appropriate facility is reached.

The pathways are also heavily influenced by the ability to receive adequate care at the facility. BEmONC facilities are consistently unavailable in many post-conflict countries, such as Sierra Leone [43], Liberia [44] and other developing countries [45]. However, the availability of comprehensive facilities is usually not a major concern. The recommended minimum number of CEmONC facilities is one facility per 500,000 of population [46]. While most countries, even the least developed, meet this recommendation, the main issue is the quality and the effectiveness of care available in these facilities, including the ability to provide the full range of CEmONC functions [45, 47]. Additionally, studies evaluating the quality of maternal healthcare often do not report facility-side barriers in sufficient detail [48]. It has been argued that the focus on patient-side delays may mask the fact that many maternal health facilities are unable to cope with obstetric complications in an effective manner [48]. The pathways described in this study clearly indicate that women seeking CEmONC in Renk hospital endure significant delays before receiving definitive treatment.

In an ideal situation, when a pregnant woman who is in labour decides to seek care, she should have access to a healthcare facility within less than five kilometres that provides BEmONC [46]. This healthcare facility should provide assistance in vaginal delivery, the removal of placenta and its retained products, and provide injectable antibiotics, oxytoxics and anticonvulsants. If the woman is in need of an emergency caesarean section or blood transfusion, the healthcare facility staff should be able to make an emergency referral to a facility that can provide this CEmONC [46, 49]. The common strategies to overcome the difficulties of physically accessing available services are an emergency transport fund, provision of immediate alternative forms of transportation, and provision of maternity waiting homes [50]. BEmONC facilities should provide BEmONC services. However, this is not always the case as illustrated in our study. Additionally, access to CEmONC is determined by: (1) the functionality of the first point of healthcare service to provide BEmONC services, and; (2) the competencies and ability of providers to make an appropriate decision to refer the patient on time, without any delay, to a functioning and appropriate healthcare facility.

Poor referral systems and extensive pyramidal and multilevel referral structures delay treatment and put patients at risk [51]. Studies conducted in Africa have shown that the majority (61–82%) of women who deliver at hospitals with childbirth facilities are self-referred [52–54]. The bypassing of referral structures, which might be initiated by the user or a lower-level healthcare provider [55], reflects a lack of confidence in the services and referral process [17]. Self-referral to hospital and bypassing referral structures in settings with difficult transportation and weak health systems has been argued to be the most realistic, speediest and safest option for women with obstetric complications [17]. Evidence from Sri Lanka and Malaysia show that bypassing the level of BEmONC increased access to a professional cadre of birth attendants and to hospitals, and led to a reduction of MMR [45, 56]. However, self-referral and underutilisation of lower-level facilities can result in congestion of hospitals, overcrowding, and poor quality of care, which will lead to an increase in the maternal mortality ratio (MMR) [57]. Other countries have prioritised an investment in hospitals over BEmONC facilities trying to increase institutional deliveries. As our research indicates, decision-making processes are not individual based and delays in decisions made about seeking and reaching care have a considerable influence on accessing care.

TBAs in South Sudan play an important role in maternal healthcare and influence access to service. The Reproductive Health Strategic Plan 2013 – 2016 abolished the training of new TBAs and has changed TBAs roles into community health workers who are not allowed to perform deliveries [25]. However, given the shortage of skilled birth attendants in South Sudan, ensuring skilled attendance at all births is neither feasible nor achievable in the short term. A range of mechanisms for TBA training and integration of their services into comprehensive reproductive healthcare services [58] are reported in the literature. Some of these may have potential to address the extreme human resources shortfalls in remote areas in South Sudan.

The context in which the health system in South Sudan operates is very complex and characterised by fluidity of the political situation, changes in the conflict status, and repeated surfacing of internal divergence. The political crisis combined with the economic and environmental crisis hampers investment in health. The current reproductive health policy recognises this situation, but the lack of investment and in particular the lack of trained skilled birth attendants is a major limiting factor in policy implementation. The quality of health facilities at community level and the competency of healthcare providers are critical. Non-functioning or partially functioning healthcare facilities need to be made functional and supplied with competent healthcare staff and resources to provide the required standard of care needed. This is clearly illustrated in the cases described above.

There are some limitations to our study that warrant caution in generalising findings. When the research team started this study, South Sudan was part of Sudan and was described as “post-conflict”. Shortly after our study started, South Sudan began preparing for the referendum. By the time the research team finished the fieldwork, South Sudan was already an independent country. At the time of writing, conflict has recommenced in South Sudan, and the outcome is unpredictable. This constantly changing situation puts some limitations on the applicability of the research findings. Conflict is constantly in flux; as conditions change, local people and health system actors change and adapt in dynamic and sometimes unpredictable ways. Therefore, a comprehensive description cannot be formulated about a particular setting. However, concepts developed and understanding can be transferable. Attempts to extrapolate the findings of this research in terms of understanding of access to healthcare must take the dimensions of context and conflict status into account. The broader research study described in this article and other articles [31–33] in which these pathways were identified may assist with applying the findings to other settings.

## Conclusions

According to the South Sudan Basic Package of Health and Nutrition Services [24], essential obstetric care (EOC) is part of Integrated Reproductive Health Services (IRHS) and is modelled around the establishment of a readily accessible quality EmONC. Additionally, IRHS includes Women’s Reproductive Health Services, Adolescent Sexual and Reproductive Health Services and Men’s Reproductive Health Services [24]. However, BEmONC is not yet available in many parts of South Sudan, and most of the existing healthcare facilities are in a poor functioning state. They lack basic equipment and most of the maternal and neonatal health workers lack the necessary skills to perform simple life-saving and nursing procedures [59, 60].

A pregnant woman needs to reach the appropriate healthcare facility as soon as possible in order for her life to be saved. Four pathways to referral care were identified in this research: ‘late referral, ‘zigzagging referral’, ‘multiple referrals’ and ‘bypassing non-functioning facilities’. Our findings indicate that maternal and neonatal outcomes are better where there is no facility available at community level, than when the woman accesses a non-functioning facility, and the absence of a healthcare provider is better than the presence of a non-competent provider. Women who bypassed non-functioning facilities and went direct to appropriate facilities survived. Visiting non-functioning or partially functioning healthcare facilities on the way - serviced by non-competent providers - places the woman at greater risk of dying. Non-functioning facilities and non-competent providers are likely to contribute to the deaths of women.

## Additional files

Additional file 1: Questions for Critical incident technique interviews. This additional file provides the questions used for Critical incident technique interviews. (DOCX 18 kb)

Additional file 2: Questions for stakeholder interviews. This additional file provides the questions used for stakeholder interviews. (DOCX 18 kb)

## Abbreviations

ANC: Antenatal care
BEmONC: Basic emergency obstetric and neonatal care
CEmONC: Comprehensive emergency obstetric and neonatal care
CIT: Critical incident technique
ICD: International classification of diseases
MA: Medical assistant
MD: Maternal deaths
MNM: Maternal near miss cases
TBA: Traditional birth attendant
