# Supplementary material for: Patterns and determinants of pathways to reach comprehensive emergency obstetric and neonatal care (CEmONC) in South Sudan: qualitative diagrammatic pathway analysis
Source: BMC Pregnancy Childbirth. 2017 Aug 29;17:278. doi: 10.1186/s12884-017-1463-9 (PMC5576292; doi:10.1186/s12884-017-1463-9)
Supplement: Supplementary file 1 — Questions for Critical incident technique interviews. This additional file provides the questions used for Critical incident technique interviews. (DOCX 18 kb) [file 12884_2017_1463_MOESM1_ESM.docx]

## ****Additional file 1: Questions for Critical incident technique interviews****

**Introduction**

1. reintroduce the study
2. Ice-breaking questions and getting to know the interviewee.
3. Personal and demographic data
4. Background information about the family
5. Details information about the respondent and their relationship to the deceased mother in case of maternal deaths.

**Body**

1. The main question is ‘what happened?’
2. Enable the interviewee to speak about the event as much as possible.
3. Return to the beginning of the story and ask follow-up and probing questions in order to get as much detailed data as possible.

**Specific questions**

Depending on the specific event and interviewee, ask the following questions:

1. Past obstetric history
2. Previous similar experiences
3. Culture, beliefs and choice of health-seeking behaviours

**End**

1. Ask about opinion for future resolutions and recommendations
2. Revisit the answers of some questions that were not clearly answered.
3. Thank Interviewees for their full participation and help.
