# Supplementary material for: Patterns and determinants of pathways to reach comprehensive emergency obstetric and neonatal care (CEmONC) in South Sudan: qualitative diagrammatic pathway analysis
Source: BMC Pregnancy Childbirth. 2017 Aug 29;17:278. doi: 10.1186/s12884-017-1463-9 (PMC5576292; doi:10.1186/s12884-017-1463-9)
Supplement: Supplementary file 2 — Questions for stakeholder interviews. This additional file provides the questions used for stakeholder interviews. (DOCX 18 kb) [file 12884_2017_1463_MOESM2_ESM.docx]

## ****Additional file 2: Questions for stakeholder interviews****

**Background information**

- Could you please introduce yourself?
- For how long have you been working as a (..........)

**Introduction**

1. What are the main problems facing women in South Sudan?
2. What are the main problems facing women’s health in South Sudan?
3. What is your evaluation of the health of pregnant women in Renk and in South Sudan?

**Acceptability**

1. In your opinion, to what extend do women utilise the health services in Renk?
2. In your opinion, what are the barriers that prevent women from utilising the health services in Renk?
3. To what extent do women accept the existing maternal health services?
4. Are there beliefs or cultures that might have an influence on pregnant women’s access to health facilities and use of it?
5. What is the role of the communities in the existing maternal health services?
6. To what extent do communities engage in maternal health services?

**Affordability**

1. What is the cost associated with the childbirth?
2. Do you think that the cost of maternal care services prevents women from fully utilising the services?
3. Do you think that families in Renk can afford the cost associated with receiving maternal healthcare?
4. Do you think that families in Renk are willing to pay for the cost associated with receiving maternal healthcare?

**Accessibility**

1. How easy is it for the people to reach the health services in Renk County?
2. What are the barriers that make it difficult for the people to reach health services in Renk County?

**Availability**

1. What is your evaluation of the geographical distribution and coverage of the healthcare facilities in Renk County?
2. What is your evaluation of the availability and quality of healthcare professionals working in maternal health?
3. Is there a budget and are there means to provide good maternal health in Renk County?

**Quality:**

1. What is your evaluation of the quality of maternal health services at the level of the midwives, health centres and hospitals?
2. What is your evaluation of the ability of the healthcare professionals in hospital to diagnose and treat pregnancy and childbirth problems?
3. What is your evaluation of the ability of trained village midwives to diagnose and treat pregnancy and childbirth problems?
4. What is your evaluation of the ability of traditional birth attendants to diagnose and treat pregnancy and childbirth problems?
5. To what extend are the people in Renk County satisfied with the health services?

**Understanding the context**

1. Do you think the health system and people responsible for the health services are aware of the beliefs, culture and barriers that might prevent the southern women from accessing health services?
2. To what extent is this awareness and understanding being useful in planning for maternal health services in Renk County?
3. How are the decisions, policies and plans for maternal health in Renk County made?
4. In your opinion, what are the solutions and strategies to provide better maternal healthcare at the levels of villages and city?
5. In your opinion, what are the solutions to making women in South Sudan fully utilise the health services of the midwives and of the hospitals?
